# Nanomaterials as drug delivery agents for overcoming the blood-brain barrier: A comprehensive review

**DOI:** 10.5599/admet.2043

**Published:** 2023-10-31

**Authors:** Mangesh Kulkarni, Krishi Patel, Ayush Patel, Swayamprakash Patel, Jagruti Desai, Mehul Patel, Umang Shah, Ashish Patel, Nilay Solanki

**Affiliations:** 1 Department of Pharmaceutical Technology; L J Institute of Pharmacy; L J University; Opp. Kataria Motors; Sarkhej-Gandhinagar Highway-382210, India; 2 Department of Pharmaceutics, Ramanbhai Patel College of Pharmacy, Charotar University of Science and Technology (CHARUSAT), CHARUSAT Campus, Changa 388421, India

**Keywords:** CNS, drug delivery, nanoparticle, liposomes, dendrimer, safety and regulation

## Abstract

**Background and Purpose:**

The blood-brain barrier (BBB), a critical interface of specialized endothelial cells, plays a pivotal role in regulating molecular and ion transport between the central nervous system (CNS) and systemic circulation.

**Experimental Approach:**

This review aims to delve into the intricate architecture and functions of the BBB while addressing challenges associated with delivering therapeutics to the brain. Historical milestones and contemporary insights underscore the BBB's significance in protecting the CNS.

**Key Results:**

Innovative approaches for enhanced drug transport include intranasal delivery exploiting olfactory and trigeminal pathways, as well as techniques like temporary BBB opening through chemicals, receptors, or focused ultrasound. These avenues hold the potential to reshape conventional drug delivery paradigms and address the limitations posed by the BBB's selectivity.

**Conclusion:**

This review underscores the vital role of the BBB in maintaining CNS health and emphasizes the importance of effective drug delivery through this barrier. Nanoparticles emerge as promising candidates to overcome BBB limitations and potentially revolutionize the treatment of CNS disorders. As research progresses, the application of nanomaterials shows immense potential for advancing neurological therapeutics, albeit with careful consideration of safety aspects.

## Introduction to blood-brain barrier

### Background of blood-brain barrier

**Figure 1.**
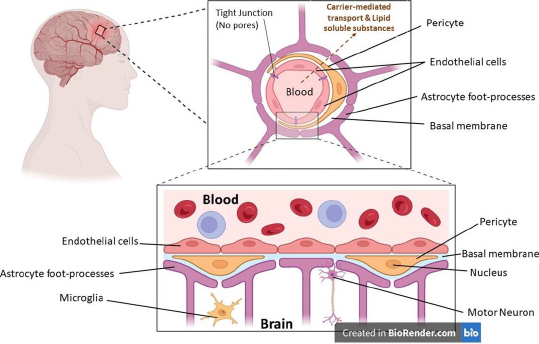
Pictorial representation of the blood-brain barrier

The blood-brain barrier (BBB), formed by the endothelial cells that line cerebral micro vessels, plays a crucial part in maintaining a carefully regulated milieu for dependable neuronal signalling. This is because the BBB is formed by the endothelial cells that line cerebral micro capillaries. Much research is being done on how brain micro vessels, astrocytes, and neurons can work together to produce functional neurovascular units, and new studies have shed light on how important brain endothelial cells are in this modular organisation. The anatomical representation of the blood-brain barrier (BBB) is shown in Figure 1, where various parts of the BBB have been depicted.

The presence of BBB was first found by Paul Ehrlich and confirmed by Edwin Goldmann. BBB isolates foreign particles in the blood from the central nervous system (CNS) and thus plays a crucial role in the protection of the CNS [1]. The CNS is composed of two vital segments of the human body, namely the human brain and the spinal cord. The BBB and blood-cerebrospinal fluid barrier (BCB) cover these two.

The BBB functions as an extremely selective permeable barrier that mediates the transport of ions and molecules between the systemic circulation and the central nervous system [2]. The blood-brain barrier comprises two distinct cellular components: endothelial cells (ECs) and mural cells (MCs). Endothelial cells, which constitute the inner lining of blood vessels, are modified simple squamous epithelial cells originating from the mesoderm. The ECs exhibit a microvascular phenotype and display a thickness that is 39% lower than that of the muscular ECs [3].

The ECs of the blood-brain barrier exhibit distinctive features, enabling them to govern the transport of ions, molecules, and cells across the interface, separating the bloodstream and the brain. The observed phenomenon exhibits a low incidence of cytopempsis, a transcellular transport mechanism facilitating the intracellular movement of diverse macromolecules. The intercellular tight junction (TJ) proteins expressed by these endothelial cells play a crucial role in impeding paracellular transport [4].

**Figure 2.**
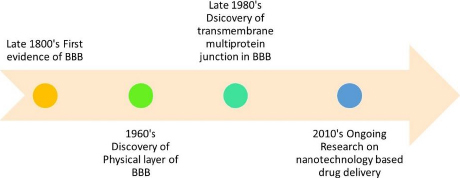
Roadmap of discovery and development of blood-brain barrier

Mural cells, herein referred to as MCs, comprise smooth muscle cells enveloped by large vessels and pericytes. Pericytes are recognised to harbour contractile proteins and illustrate an impulse to contract, thereby regulating the diameter of the capillaries [3]. The BBB is comprised of a series of uninterrupted ECs that are reinforced by tight junctions (TJs), adherent junctions (AJs), and gap junctions (GJs). The TJs are a crucial component of the BBB, providing increased trans-endothelial resistance within the BBB [5]. The present study elucidates that the aforementioned intercellular junctions function as drug monitoring agents via cellular tethering, resulting in intercellular space occlusion [6]. The first evidence of the presence of BBB was seen in the late 1800’s. The discovery of the physical layer of BBB was made in the 1960s. The transmembrane multiprotein junction was discovered in the era of 1980s. Figure 2 represents the roadmap to the discovery and development of the BBB.

### Challenges in drug delivery to BBB

**Figure 3.**
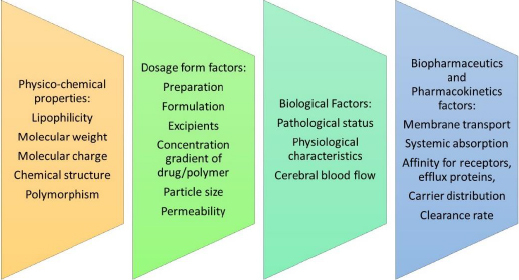
Factors affecting drug delivery across BBB

At present, there exists a global population of approximately 1.5 billion individuals who are afflicted with various CNS disorders at any given point in time. The primary impediment to effective drug delivery to the brain is the BBB and the associated enzymatic activity that regulates the influx of molecules into the CNS [7]. Figure 3 elaborates on the factors that affect drug delivery across the BBB.

### Importance of nanoparticles in overcoming BBB limitations

The administration of pharmacological agents to the brain can be achieved through a multitude of delivery methods. Initially, the administration of pharmaceutical agents can be achieved via localised delivery. The administration of therapeutic agents in a localised manner can be achieved through the utilisation of catheter-based injection or convection-enhanced delivery systems. Biodegradable implants have the potential to provide sustained drug release capabilities [8]. The aforementioned procedures are inherently invasive due to their surgical nature. This area presents a potential opportunity for localised drug delivery, potentially representing a novel frontier in the field. An alternative method for drug delivery involves administration via the intranasal route.

Upon arrival at the nasal cavity, the transportation process may proceed via the olfactory and trigeminal pathways, ultimately leading access to the CNS [9]. The non-invasive and innovative approach has garnered the interest of numerous researchers. The nasal administration modality exhibits certain limitations, such as the lack of consistency in the administered dosage of the formulation [10]. One potential avenue for improving drug delivery involves augmenting the capacity of drugs to traverse the BBB through modifications to their molecular architecture or the implementation of prodrug strategies.

Levodopa, a prodrug for dopamine, is a notable illustration of this approach and is employed to treat Parkinson's disease. The feasibility of this option is often constrained by the limitations associated with molecular modification or fabrication. In addition, the potential for reversible disruption may be explored through the utilisation of hyperosmolar agents such as mannitol injection [11] or by using physical methodology such as ultrasound [12], thereby presenting a viable alternative. However, the disruption of the BBB can result in considerable harm to the brain, as the BBB serves as a crucial protective barrier for the human brain [13].

Various techniques are available to explore drug delivery methods to the brain. Localized drug delivery through catheter-based injection, convection-enhanced delivery systems, and biodegradable implants offer potential advantages. However, it's important to acknowledge the invasiveness associated with these surgical procedures. Despite this, the potential for localized drug delivery in this domain holds promise and may signify a significant advancement in the field.

An intriguing non-invasive alternative is intranasal drug administration, which has attracted considerable interest among researchers. Although it has challenges, notably in maintaining consistent dosages, the potential for delivering drugs to the CNS through this route is notable. One key area of interest is the utilization of nanoparticles to overcome the BBB limitations. Modifying drug molecular structures or employing prodrug strategies, as demonstrated by levodopa, offers exciting potential for improving drug delivery to the brain. However, it's essential to recognize the constraints and potential risks associated with these strategies, particularly in terms of the BBB's protective function for the brain.

Additionally, considering reversible disruption of the BBB using agents like mannitol injection or physical methodologies such as ultrasound represents an intriguing direction for research. Nonetheless, the balance between enhancing drug delivery and preserving the BBB's protective role is a critical consideration in pursuing innovation within the field.

## Anatomy of BBB

### Structure and composition of BBB

BBB is comprised of various cellular components, including endothelial cells (ECs), tight junctions (TJs), and adherent junctions (AJs). These components regulate and facilitate the emergence and maintenance of the BBB through the control of EC proliferation, migration, and vascular branching within the brain. The extracellular matrix, known as the basement membrane, is essential for providing structural support in the vicinity of pericytes and endothelial cells. The basal lamina exhibits continuity with the plasma membrane of the astrocyte end-feet [14].

#### Endothelial cells (ECs)

The mitochondrial content of BBB ECs is comparatively higher than that of the peripheral vasculature. These cells also display minimal pinocytic activity and are devoid of fenestrations. The limited paracellular permeability of the endothelial cell layer is justified due to the presence of two intercellular molecular binding mechanisms. The present study aims to investigate the potential relationship between TJs and AJs [15].

#### Astrocytes

Astrocytes, an assortment of glial cells, are imperative in the upkeep and safety of neurons. They achieve this by regulating neurotransmitter and ion concentrations, which helps to maintain the homeostatic balance of the neural microenvironment. Additionally, astrocytes modulate synaptic transmission and regulate immune reactions, further contributing to their important role in neural function [16] by interacting with endothelial cells through their end feet projections that encircle the basolateral side of cerebral capillaries, astrocytes exhibit a profusion of slender projections that sheath cerebral vasculature and enwrap both individual and clustered synaptic junctions [17]

Astrocytes are the predominant cell type found in the CNS and are known to participate in a variety of physiological and biochemical processes [18]. The functions mentioned earlier encompass (1) the emergence of distinct compartments within the parenchyma, (2) the preservation of extracellular ionic homeostasis, (3) regulation of pH, (4) the facilitation of uptake of neurotransmitters, and (5) the mediation of neuronal signals to the vasculature.

#### Pericytes

While Eberth initially reported their existence in 1871, the credit for the discovery of pericytes is generally attributed to Charles-Marie Benjamin Rouget, a French researcher who, two years later, identified a group of contractile cells that encircle the endothelial cells of minor blood vessels.

Pericytes, also referred to as Rouget cells or mural cells, have been observed to be present in nearly all capillaries, in addition to small arterioles and venules. The aforementioned entities are diminutive cellular structures in the extravascular space, interposed amidst the endothelial monolayer and the parenchymal tissue. The basal lamina serves as a partition between the parenchyma and the pericyte and endothelial cells. The morphological characteristics of pericytes exhibit variability based on their location along the arterio-venous axis and the vascular bed. Typically, pericytes extend primary projections in both directions along the vessel from the soma. These projections subsequently give rise to secondary and tertiary processes that envelop the vessel [19]. Pericytes maintain BBB integrity [20], regulate angiogenesis (Microvascular remodelling), regulate phagocytosis [21], maintain blood flow and capillary diameter, regulate leukocyte trafficking into the brain and maintain multipotent stem activity.

Pericytes are a crucial component of the cerebral capillary and exhibit varying densities across distinct vascular regions. The cells exhibit high prevalence within the central nervous system, with a notable concentration observed within the retinal tissue [22].

### Functions of BBB

The human BBB has several functions that are pivotal in maintaining neuronal health. The major functions have been explained below:

#### Maintaining ionic homeostasis and brain nutrition

The BBB maintains a regulated microenvironment by means of selective ion channels and transporters that ensure the ideal ionic composition for efficient neural and synaptic signalling processes. The maintenance of potassium levels in cerebrospinal fluid (CSF) and interstitial fluid (ISF) is observed to be approximately 2.5-2.9 millimolars (mM). The plasma concentration, when compared, exhibits an approximate value of 4.5 mM.

However, it is noteworthy that fluctuations in potassium plasma levels may arise due to various factors such as exercise, meal consumption, experimental imposition, or pathological conditions [23]. The presence of distinct ion channels and transporters at the BBB creates an ideal milieu for the maintenance of synaptic and neural function.

#### Neurotransmitter level regulation

The transportation of neurotransmitters from the brain to the bloodstream predominantly relies on sodium^-^coupled and sodium^-^independent amino acid transporters. The BBB restricts the entry of certain amino acids, such as the neurotransmitters glutamate and glycine, while facilitating the exit of numerous other indispensable amino acids [24].

#### Prevention of macromolecule entry into the human brain

Under normal body conditions, the BBB prevents the entry of macromolecules. Plasma proteins like albumin, prothrombin and plasminogen could harm the nervous tissues, triggering cellular activation, which might lead to apoptosis. There is an evidential presence of various activators in the human brain and within the CNS; one of these is Factor X_a_. Factor X_a_ is responsible for the conversion of prothrombin to thrombin, or tissue plasminogen activator, which converts plasminogen to plasmin. The resulting proteins, plasmin or thrombin, can attach to their receptors in the human brain tissue and trigger or cascade sequences of events like seizures, glial activation, glial cell division, and scarring followed by cell death. Thus, it is evident that the BBB is a “gatekeeper” for the human brain and the CNS system, which selectively allows the entry of only those seemingly beneficial components.

#### Protection against neurotoxins

Numerous neurotoxins derived endogenously from metabolites or proteins or exogenously from xenobiotics are often detected circulating in the human blood. The ATP-binding cassette (ABC energy-dependent efflux transporters) occupies the BBB luminal surface [25]. The CNS of an adult individual has limited regenerative ability if damaged by any means. From the birth of the human being, neuronal cell death is inevitable, but this occurs comparatively slowly. The presence of neurotoxins would trigger this pace and would turn prematurely debilitating [26].

## Nanomaterials for drug delivery across BBB

As previously stated, the transportation of drugs to the brain can be achieved through either invasive or non-invasive means. The utilization of nanomaterials as a drug delivery system offers a versatile approach to administering drugs, as the methodology can be tailored to meet the specific requirements of the drug delivery process.

This involves designing, fabricating, and characterizing such structures, with the ultimate goal of comprehending and leveraging their unique properties and functionalities. Nanomaterials can potentially infiltrate the human body through various pathways, including the respiratory, dermal, gastrointestinal, and parenteral routes. The need to mould pharmaceutical active ingredients into nanomaterials stems from their remarkable capacity to encapsulate, release, and selectively target drugs to virtually any organ within the body [27]. Numerous investigations have documented the efficacious transport of hydrophilic and hydrophobic drugs, proteins, biological macromolecules, and even vaccines using nanoparticles as transport systems [28]. Nanoparticles (NPs) possess the potential to be modified in order to incorporate a range of features, including but not limited to:

- biocompatibility,
- reduced toxicity,
- the capacity to bind and transport multiple loads,
- the ability to protect therapeutics from in vivo degradation,
- the ability to regulate the release of therapeutics over extended periods, and
- the ability to traverse the BBB.

**Figure 4.**
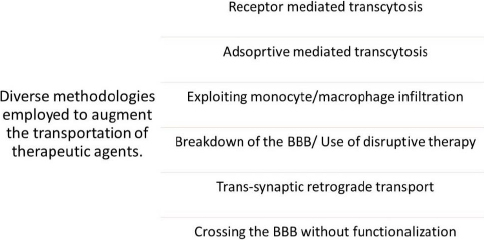
Diverse methodologies employed for the transportation of therapeutic agents.

Various nanomaterials have the capability to encapsulate, absorb, attach, or trap drug moieties. Nanoparticles or nanomaterials exhibit a noteworthy advantage in that they can be employed for intravenous delivery in preference to other microparticles [29]. The diverse mechanisms by which a pharmaceutical agent can be administered to the human blood-brain barrier (BBB) comprise the subsequent approaches mentioned in Figure 4. Figure 4 illustrates the diverse methodologies employed for the transportation of therapeutic agents across BBB.

The objective is to induce the opening of TJs or elicit localized toxic effects, which may lead to an augmented permeability of the BBB. This, in turn, would make it possible for the infiltration of drugs or drug-loaded nanomaterials into the CNS [30]. The transcytosis mechanism enables passage through the ECs [31]. The ECs have the ability to internalize the particles through the process of endocytosis, which is then followed by the release of their intracellular cargo [32]. Table 1 depicts techniques involving invasive and non-invasive routes to deliver therapeutics to the human brain.

**Table 1.** List of invasive and non-invasive techniques for drug delivery to the human brain

Techniques	Methods/Mode	Strategy
Invasive	BBB disruption techniques	Osmotic BBB disruption strategy, Biochemical BBB disruption strategy, Ultrasound mediated BBB disruption strategy [33]
Other routes for CNS drug delivery	Other routes	Olfactory pathway, Trigeminal pathway, Intranasal delivery, Iontophoretic delivery
Non-Invasive	Chemical	Lipophilic analogs, Prodrugs, Chemical drug delivery system, Molecular packaging, Receptor/Vector mediated delivery of chimeric peptides
Non-Invasive	Biological	Viral vectors, Cell penetrating peptide-mediated drug delivery, Nanospheres, Nano capsules
Non-Invasive	Colloidal drug carriers	Solid Lipid Nanoparticles (SLNs), Dendrimers, Single and multi-walled carbon nanotubes [34]
Non-Invasive	Pharmacological	Intraventricular/Intrathecal/Interstitial delivery, Biological tissue delivery, Convection-enhanced delivery [35]

### Ideal properties of nanomaterials

**Figure 5.**
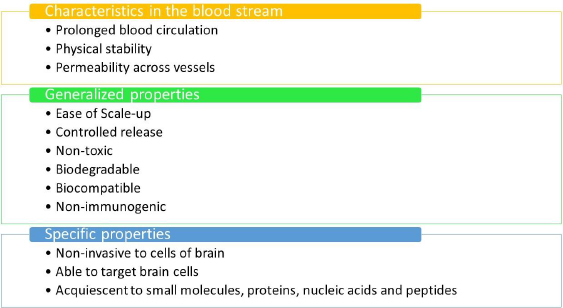
Ideal properties of nanomaterials to deliver drugs across BBB

Figure 5 outlines the optimal prerequisites for a nanomaterial to be considered suitable for the delivery of drugs to the human brain. Figure 5 mentions the ideal prerequisites for nanomaterials to be used in therapeutics.

### Nanomaterials explored for drug delivery to the brain

In 1961, Muller and Gasco rendered the discovery of lipidic nanoparticles. Lipidic nanoparticles (LNs) are a class of nanotechnology drug delivery systems that encompass solid lipid nanoparticles (SLNs) [36], nanostructured lipid carriers (NLCs), and liposomes [37].

**Figure 6.**
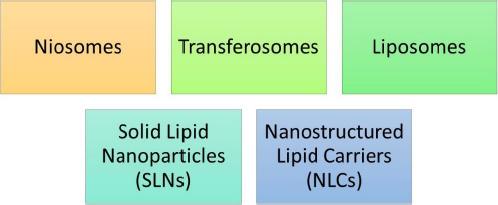
Several types of lipidic nanoparticles

LNs exhibit a number of benefits over colloidal nanoparticles, including improved drug entrapment, extended drug release, amplified physical and chemical stability, and superior incorporation or encapsulation of lipophilic drugs within the core of SLNs [38] and NLCs [39]. Figure 6 represents various types of lipidic nanoparticles. It generally encompasses SLNs, NLCs, liposomes, transferosomes and niosomes.

These nanoparticles offer a range of advantages over colloidal nanoparticles. Firstly, they demonstrate improved drug entrapment, ensuring a higher drug payload can be incorporated. Secondly, lipid nanoparticles enable extended drug release, ensuring a more sustained and controlled drug release, which is crucial for effective therapeutic outcomes. Additionally, they provide enhanced physical and chemical stability, making them a reliable choice for drug delivery systems. Notably, lipid nanoparticles excel in incorporating or encapsulating lipophilic drugs within the core, making them efficient carriers for these types of drugs.

SLNs and NLCs are specifically highlighted within this category of nanoparticles. These subtypes offer versatile drug delivery options due to their solid lipid matrix that can effectively entrap drugs. Liposomes, transferosomes, and niosomes are also part of this category and contribute to the diverse array of lipidic nanoparticles available for drug delivery.

The main lipid-based carriers include:

- Niosomes: niosomes are lamellar, self-assembled structures comprising non-ionic surfactants and cholesterol [40].
- Transferosomes: they are identical to niosomes and liposomes and they also have a lipid bilayer developed by a lipid matrix that is stabilized by the usage of several surfactants.
- Liposomes: the liposomes are bilayer vesicles consisting of phospholipids [41,42].
- Solid lipid nanoparticles (SLNs): the SLNs contain a core of solid lipid that helps to encapsulate the drug [43].
- Nanostructured lipid carriers (NLCs): The NLCs have a core of liquid lipid interior to the solid lipid phase [44].

### Strategies to modify drug release through BBB penetration

#### Physiological transport mechanism

##### Receptor-mediated transportation (RMT)

Receptor-mediated transcytosis encompasses the intricate process by which nanoparticles effectively interact with specific receptors situated on the apical surface of endothelial cells, facilitated by a diverse array of ligands. This interaction subsequently triggers the internalisation of the nanoparticles via endocytosis. In a manner akin to adsorptive-mediated transcytosis (AMT), the process of intracellular transport vesicle formation involves the inward folding of the cell membrane, facilitating the transportation of NPs towards the basolateral surface [45]. NPs may be conjugated into organic or synthetic ligands, and RMT controls the homeostasis of nutrients, including iron, insulin, and leptin. These ligands serve as NP's "Trojan horses" to enter the brain. RMT makes it possible to precisely target tumour cells and brain tissue [46]. By developing ligands that can bind to receptors on the apical surface of BBB endothelial cells as well as target tissue, such as transferrin and low-density lipoprotein receptors, which are found at the BBB and are overexpressed in cancer cells, NPs may be directed to particular brain areas [47]. Liang [48] *et al.*, for instance, established in mice that the chemotherapeutic vincristine sulphate may specifically bind the LDL receptor and target glioma cells via conjugating LDL (low-density lipoprotein) particles. However, excessive avidity of NPs may prevent them from dissociating from their receptor and keep them attached to endothelial cells, which is a significant problem when designing NPs and conjugating ligands [47]. In brain cancer cells, doxorubicin-loaded polysorbate 80-coated NPs greatly increased caspase-3 expression and death, which inhibited metastasis and prolonged survival [49]. Receptor-mediated transportation (RMT) is a technology that enables precise targeting of brain tissue and tumour cells, as well as the capacity to maintain nutritional homeostasis and employ ligands as "Trojan horses" for nanoparticle entry into the brain [50]. Despite the benefits of using RMT to deliver drugs via the BBB, certain disadvantages need to be considered. One drawback is the possibility of excessive avidity, resulting in NPs remaining attached to endothelial cells instead of detaching from their receptors, leading to inefficient drug delivery. This emphasizes the importance of careful design and ligand conjugation strategies to optimize the detachment of NPs from receptors [51]. Additionally, the design and optimization of RMT systems can be complex, requiring meticulous selection and conjugation of ligands to ensure proper ligand-receptor interactions.

##### Transporter-mediated transport

The BBB is a strongly expressed transporter of nutrients that the brain needs. Researchers have coupled glutathione, an endogenous tripeptide with antioxidant characteristics, onto liposomes to transport multiple medications to the brain. Li. [52] *et al.* created a number of bis-quaternary ammonium compounds with high affinity for the choline transporter, and an in vitro investigation found that modification with a choline derivative allowed for more effective absorption by BCEC in comparison to untreated dendrimers. The same drug delivery method was also employed to administer the MRI contrast agent Gd, DNA, and doxorubicin, demonstrating good brain-targeted delivery effectiveness [49]. Transporter-mediated transport for medication administration across the blood-brain barrier offers various advantages. By boosting absorption and transport, it enhances medication delivery to the brain, enhancing therapeutic effectiveness. It also allows for targeted distribution by utilising transporters that are abundant on the BBB [53]. However, there are certain challenges associated with transporter-mediated transport, including the complexity of system design and optimisation, the limited availability and selectivity of some transporters, and the possibility of off-target effects due to expression in other tissues or organs [54]. Overall, transporter-mediated transport has the potential to improve brain drug delivery, but careful thinking and additional study are required to overcome these obstacles and maximise its potential [55].

##### Adsorptive-mediated transportation

NPs are often transported across the BBB through adsorption or receptor-mediated transcytosis in endothelial cells [56]. Adsorptive-mediated transcytosis (AMT) is a mechanism that results in vesicle production and cell membrane invasion as a result of connections between nanoparticles and the endothelial plasma membrane. Endosomes are created to transfer nanoparticles directly to the basolateral surface, lysosomes for degradation, or the apical plasma membrane [57]. Adsorptive-mediated transcytosis (AMT) is a non-receptor-mediated mechanism for nanoparticle (NP) transport across the blood-brain barrier (BBB). It provides an improved channel for delivering NPs to the brain, allowing therapeutic, imaging, and diagnostic nanoparticles to be delivered. Electrostatic interactions between cationic NPs and anionic endothelial cell membranes frequently promote NP binding, internalisation, and transit across the BBB [58]. However, due to its non-specific nature, AMT has restrictions such as a lack of selectivity, intrinsic toxicity, a limited capacity for transporting larger NPs, and competitive interactions with anionic molecules or cell membranes, which can restrict its usefulness [59].

##### Cell-penetrating peptide (CPP)-related transport

Another method involves the use of cell-penetrating peptides to improve CNS delivery [60]. CPPs are made up of a series of extremely basic amino acids that give the peptide a positive charge. They interact with the cell surface through a receptor-independent manner. CPPs can also transport molecules linked to them through the cell membrane, the cytoplasm, and the nucleus. The effect is unaffected by cell type. CPPs were found in 1988. in HIV and are known for their capacity to enhance transport across cell membranes [61]. The BBB, being a negatively charged membrane, exhibited attraction for CPPs. Qin *et al.* demonstrated that modifying transactivating-transduction (TAT) onto liposomes (TAT-liposomes) might increase accumulation in the brain by 2.54-fold over unmodified liposomes [62]. TAT protein is capable of migrating from quiescently infected cells to cells that are uninfected and initiating viral replication. TAT peptide is a short polypeptide of 86 amino acid residues and a cysteine-rich region. TAT peptide has been shown to permeate the cell membrane in a receptor and transporter-independent manner. Endocytosis and pinocytosis are the basic processes postulated to explain TAT peptide cellular absorption. TAT conjugation was used to help transfer biomacromolecules over the BBB. Another investigation found that TAT-conjugated chitosan NPs improved the transport of genes to the brain [63]. Gd3 and doxorubicin may be delivered to gliomas more effectively by TAT-modified gold nanoparticles than by Gd3 and free doxorubicin [64]. Additional CPPs, including octa arginine, have been proven to have the ability to target the BBB, and it has been shown that the effectiveness of brain targeting is directly correlated with CPP positive charge [65]. The chance of a drug-related adverse effect increased as a result of CPP change since it enhanced BBB penetration and accumulation in other organs, including the liver and spleen [66]. Sharma *et al.* developed the bi-ligand Tf-PR by conjugating Tf with poly-L-arginine. Tf-PR-liposomes had much higher cellular uptake in vitro than Tf-Liposomes and PR-Liposomes. After 8 hours of incubation, the transfer percentage of Tf-PR-liposomes was 19%, significantly greater than that of Tf-liposomes (13%) and PR-liposomes (10 %) [67]. Tf-PR-liposomes had about a 2-fold greater brain targeting impact than Tf-Liposomes in vivo. As a consequence, gene expression was 1.66-fold higher in the brains of mice treated with gene-loaded Tf-PR-liposomes than in Tf-Liposomes [68]. Liu *et al.* [69] combined octa-arginine with the c(RGDfK) peptide to create the bi-ligand RRGD.

After paclitaxel loading, animals with brain tumours treated with RRGD-liposomes had median survival times of 1.33 and 1.26 times longer than those of mice treated with R-liposomes and RGD liposomes, respectively [69]. Cell-penetrating peptides (CPPs) have various benefits for delivering load inside cells. They improve cellular uptake of various cargo molecules, breaking down cellular barriers and allowing for efficient internalisation. CPPs have minimal toxicity, good biocompatibility, and the capacity to penetrate multiple cell types, making them ideal for targeted distribution [70].

Contrary to this, CPPs exhibit a deficiency in cell specificity, as they heavily rely on charge interactions. Additionally, CPPs may face challenges when it comes to traversing intracellular barriers. The transport facilitated by cell-penetrating peptides (CPPs) exhibits a size-dependent characteristic, wherein larger cargo molecules demonstrate reduced efficiency. Additionally, it is crucial to address the issues of immunegenicity and design complexity associated with CPP-based delivery systems [71].

### Temporarily open the BBB

Several physical and pharmacological approaches could be used to briefly access the BBB. Because the BBB is the primary barrier preventing compounds or NPs from entering the brain directly, temporarily opening the BBB to enlarge the pore size might allow substances or NPs to enter the brain directly.

#### Chemicals that enhance BBB permeability

The permeability of several membranes, including the mucosa, skin, and BBB, can be increased by borneol, a messenger medication often used in traditional Chinese medicine [72].

Similarly, alkylglycerols may open the BBB and promote medication transport to the brain. Modified alkylglycerol was grafted onto dextran-graft-poly(lactic acid) NPs for delivery with a focus on the brain (PLA-DEX-OX4). Compared to free dextran and unmodified NPs, PLA-DEX-OX4 penetrated the bEnd.3 monolayer more deeply. Sadly, no statistically significant difference was found, suggesting that the alkylglycerol modification's effectiveness was satisfactory [73].

The myriad advantages associated with chemical agents that enhance BBB permeability to facilitate medication delivery to the brain are manifold. The compounds possess the potential to be strategically engineered in order to selectively interact with a particular subset of cerebral cells or regions, thereby facilitating localised therapeutic efficacy while minimising the occurrence of undesirable secondary effects. The utilisation of combination treatment, wherein chemical agents are concomitantly employed with alternative methodologies, has been observed to elicit a heightened permeability of the BBB and subsequently enhance the therapeutic efficacy of the treatment regimen [74].

However, it is imperative to thoroughly evaluate certain chemicals' potential toxicity and non-specific effects due to their inherent toxicity and lack of selectivity. Due to the transient nature of enhanced permeability, it may be imperative to administer multiple doses or employ sustained-release formulations to achieve long-term benefits. The clinical translation of these pharmaceutical compounds presents considerable challenges, necessitating further investigation and refinement to address regulatory and safety considerations [75].

#### Receptor-involved changing of tight junctions

Modifying the tightness of tight junctions is a possible method for increasing BBB permeability since tight junctions are essential for preserving BBB integrity and preventing substances from entering the brain [49]. Four subtypes of adenosine receptors known as G-protein-coupled receptors are recognised: A1, A2A, A2B, and A3 [76].

A plethora of adenosine receptor agonists and antagonists exhibiting therapeutic attributes can be observed in the current scientific literature [77]. Gao *et al.* have recently proven that turning on the A1 and A2A adenosine receptors can make the BBB more permeable in vivo. One A2A agonist, Lexiscan, may enhance macromolecule penetration through the BBB.

Adenosine receptor agonists might be used to transport medications to the brain [78, 79]. The use of Lexiscan-conjugated dendrimers resulted in the production of nano agonists that improved the targeted delivery of several model medicines (NAs). Den-Reg16, which combines 16 lexiscan molecules onto one dendrimer, has a 7.7-fold higher affinity for the A2A adenosine receptor than unaltered lexiscan. In untreated bEnd.3 monolayers, ZO-1 expression was continuously aligned on the cell-cell interface in vitro; however, Den-Reg16 treatment halted expression and increased the permeability of the model drug by 17.6 times. In mice pretreated with Den-Reg16, the model drug accumulation in the brains was 6.8 and 3.6 times greater than in saline with lexiscan, respectively.

Additionally, there is an increase in brain-focused drug delivery as measured by SPECT/CT [79]. The nanoagonists were found to be promising adjuvants for enhancing BBB permeability. Tight junction transition mediated by receptors has various advantages for medication administration across the BBB. By modulating the permeability of the barrier at predetermined locations it enables the precise and targeted delivery of therapeutic agents to specific tissues or cells. Receptor-mediated modulation, owing to its inherent reversibility, affords the advantageous capacity for temporal regulation and adaptability in the realm of pharmaceutical administration [80]. Nevertheless, it is imperative to acknowledge certain constraints inherent in this approach. These limitations encompass the absence of suitable receptors within the targeted region, the potential for unintended consequences on unrelated biological pathways or tissues, the inherent variability in receptor expression across the blood-brain barrier (BBB), and the indispensable requirement for meticulous adherence to regulatory and safety protocols [81].

#### Focused ultrasound

Ultrasound has emerged as a progressively favoured modality for facilitating the permeation of pharmaceutical agents across the BBB. Microbubble-enhanced diagnostic ultrasonography (MEUS) is a non-invasive modality employed to augment the permeability of the blood-brain tumour barrier (BBTB) in individuals diagnosed with glioma. The primary objective of this technique is to facilitate the passage of therapeutic agents across the BBTB, thereby enhancing the efficacy of medication delivery. The utilisation of focused ultrasound (FUS) has demonstrated its efficacy in augmenting the transport of NP cargo across the BBB [120].

Etame *et al.* [12] used targeted ultrasound that was guided by an MRI to look at the distribution of gold nanoparticles in two hemispheres. In comparison to the control hemisphere, the concentration of gold NPs was 3.36 times greater in the sonicated hemisphere. This demonstrates how therapeutic gold nanoparticle transport into the brain may be enhanced by MRI-guided focused ultrasound. Through the use of FUS and microbubbles in conjunction with DCE-MRI, Park *et al.* [82] studied the delivery of doxorubicin.

By combining the two methods, they were able to show that the BBB and BBTB were more permeable to drugs and that the drug retention period in tissue over 24 hours was enhanced. MEUS' capacity to reduce P-gp expression over time was equally fascinating. Nonhuman primates were given FUS at different sonic pressures to explore the physiological changes in the brain caused by the FUS-induced BBB opening [83]. The buildup of drugs and NP in the brain may be accelerated by combining FUS with other targeted techniques.

When FUS and magnetic targeting were used together, magnetic NP accumulation in the brain improved 16.3 times more than when FUS was used alone [84]. Combining FUS with a targeting ligand alteration may potentially improve brain-targeted medication delivery. When combined with atherosclerotic plaque-specific peptide-1-modified liposomes, the tumor-to-contralateral brain ratios of medication rose from 2.1 to 3.8. FUS toxicity to the brain is considered minimal, and neurotoxicity was not seen in recent research [85].

Most patients showed no oedema after using FUS and microbubbles to repeatedly breach the BBB over an extended period (4 to 20 months). There should be caution while using this technology for medicinal purposes [86]. However, a substantial increase in reaction time during the task was seen on the day of FUS and microbubbles application in a neurotoxicity test using quantitative cognitive assessment of visual, cognitive, motivational, and motor function. Fortunately, the reaction time reverted to normal after 4-5 days of the surgery, proving the method's safety [87].

The utilisation of focused ultrasound has been found to yield numerous advantages, notably its non-invasive nature and remarkable capacity for precise targeting. These attributes enable the delivery of medication with exceptional accuracy or the manipulation of biological processes, all while minimising the potential harm inflicted upon adjacent tissues. The utilisation of this technology spans various domains, encompassing imaging, tumour ablation, neuromodulation, targeted gene therapy, and the promising prospect of augmenting drug administration to the cerebral region. The utilisation of this particular technique, while undoubtedly advantageous, is not without its constraints. These limitations primarily manifest in the form of restricted penetration depth and the potential for unintended consequences in non-target areas.

Consequently, meticulous strategizing and vigilant oversight are imperative to ensure optimal outcomes. The potential hazards associated with tissue injury and adverse events are key factors to be considered when considering safety measures. It is imperative to implement stringent safety protocols and exercise careful patient selection in order to mitigate these risks.

In summary, in the first approach, they highlight chemicals like borneol and alkylglycerols that can enhance BBB permeability, potentially revolutionizing drug delivery to the brain. However, they emphasize the need to carefully evaluate these chemicals due to their potential toxicity and lack of selectivity. The second approach involves modifying tight junctions using adenosine receptor agonists, showcasing the potential to selectively enhance BBB permeability for precise drug delivery. Lastly, the author underscores the promising role of focused ultrasound in facilitating drug transport through the BBB. They emphasize its non-invasive nature and precise targeting while cautioning about potential risks and the need for vigilant safety measures. Overall, the author's perspective centres on advancing drug delivery strategies to the brain, aiming for both efficacy and safety in medical applications.

### Intranasal delivery to bypass the BBB

#### Unmodified NPs

In their unaltered state, nanoparticles present a viable alternative for circumventing the BBB. The enhancement of medication administration is achieved through the inhibition of enzymatic degradation induced by nasal enzymes, the mitigation of ciliotoxicity, and the demonstrated superiority in drug delivery efficacy compared to conventional solutions. Several papers supporting this claim include Jiang *et al.* [88] nimodipine encapsulated microemulsions and Zhang *et al.* [89] H 102 peptide-loaded NPs.

Jiang *et al.* [88] found that a second-generation 1,4-dihydropyridine calcium channel blocker, Nimodipine encapsulated microemulsions, had a 13.8-fold and 159-fold greater area under the curve in the brain cerebrum and CSF when compared to IV treatment. Furthermore, it exhibited no cilia-related toxicity [90]. Similarly, Zhang *et al.* [89] H102 peptide-loaded NP formulation showed 1.6 to 2.9 fold greater concentration than H102 peptide solution [91].

The incorporation of a unique ligand into nanoparticles has been observed to enhance their targeting capabilities. Enhancements in drug penetration through the nasal mucosa have been observed, facilitating the transportation of medications into the brain and enabling more efficient delivery of therapeutic agents. Moreover, it has been observed that NPs can be meticulously engineered to selectively target distinct neuronal cells or regions within the brain, thereby facilitating the precise administration of pharmaceutical agents.

The intranasal delivery route is considered non-invasive, as it does not necessitate injections or surgical interventions. Moreover, it has been observed that the utilisation of NPs has the potential to significantly enhance the bioavailability of medications. This is primarily attributed to the ability of NPs to effectively prevent medication degradation and improve drug stability. Nevertheless, it is imperative to acknowledge the existence of various drawbacks associated with the utilisation of unmodified NP systems. These limitations primarily revolve around their inherent incapacity to effectively encapsulate a sufficient quantity of therapeutic molecules, thereby compromising their therapeutic potential. Additionally, it is crucial to consider the potential toxicity that may arise from the substances employed in the formation of these unmodified NPs. Nasal polyps exhibit rapid egress from the nasal cavity, reducing medication exposure and subsequent therapeutic efficacy. The efficacy of unmodified NPs exhibits variability contingent upon factors such as particle size, shape, and surface charge. These parameters exert an influence on the NPs' ability to traverse the nasal epithelium and ultimately access the brain. [92-94]

#### Agglutinant-mediated transport

Agglutinants can attach to saccharide groups and stimulate endocytosis, which can be used to improve intranasal medication delivery due to greater expression of saccharide groups on olfactory mucosa, such as N-acetylglucosamine and L-fucose [95]. Jiang *et al.* [96] used this approach to transfer vasoactive intestinal peptides via modified wheat germ agglutinin (WGA) onto poly(lactic-co-glycolic acid) (PLGA -NPs) (WGA-NPs). This investigation revealed that intact VIP given to the brain had 11.48 % ID h/g, which was 7.74 times higher than the VIP solution [96].

This was attributable to WGA-NPs' greater affinity and better penetration into the olfactory mucosa [97]. Similar trials with different medicines, such as *Ulex europeus* agglutinin I (UEA I), *Solanum tuberosum* lectin, and odorranalectin, yielded the same improved efficiency in drug administration [98]. A novel tiny odorranalectin-containing cubosome demonstrated promising results, including successful synthesis, effective brain transport, and pharmacodynamics evaluation in amyloid-treated rats after intranasal administration [99]. This technique has some perks, including the capacity to create agglutinnants that particularly target receptors on brain endothelial cells, allowing for precise transport of NPs to the brain. However, there are a number of disadvantages to consider.

Agglutinants may be nonselective for brain endothelial cell receptors, resulting in off-target effects or inadequate targeting. Agglutinants can also be immunogenic or toxic, eliciting immunological responses or having negative effects. The development of agglutinant-mediated transport systems is difficult and time-consuming, requiring careful agglutinant selection and conjugation to NPs [100,101].

In essence, the unmodified NPs demonstrate the potential to enhance medication administration by inhibiting enzymatic degradation, mitigating ciliotoxicity, and showcasing superior drug delivery efficacy compared to conventional solutions. The incorporation of unique ligands into NPs further enhances their targeting capabilities, allowing for precise drug penetration through the nasal mucosa and efficient delivery of therapeutic agents.

Additionally, agglutinants are a valuable tool for improving intranasal medication delivery by stimulating endocytosis through attachment to saccharide groups. They offer the advantage of specifically targeting receptors on brain endothelial cells, facilitating the precise transport of NPs to the brain. However, it is crucial to acknowledge the potential downsides, including non-selectivity for brain endothelial cell receptors, possible immunogenicity, and the intricate development process of agglutinant-mediated transport systems. The integration of agglutinants, while presenting challenges, holds promise for revolutionizing drug delivery and advancing therapeutic outcomes for brain-related conditions. The author's viewpoint underscores the need for further research and careful consideration of limitations to fully exploit the potential of these approaches.

## Nanoparticles for drug delivery across the blood-brain barrier

Various nanoparticulate drug delivery systems have been developed to enhance drug transport across the highly selective blood-brain barrier (BBB) and into the brain.

### Polymeric nanoparticles

As a result of their hydrophilic polymer covering and core-shell structure, polymeric-based NPs are an appealing option for medication administration across the BBB [102]. The development of polymer-based transport carriers frequently makes use of synthetic polymers such as polyethylene glycol (PEG), poly(lactic acid) (PLA) and poly(lactide-coglycolic-acid) (PLGA) [103, 104].

Considerable attention has been devoted to investigating the utilisation of polymeric NPs for the enhancement of chemotherapy administration to cerebral neoplasms. An exemplary illustration entails the implementation of polylactic acid nanoparticles (PLA NPs) that have been coated with transferrin (Tf) to facilitate the transportation of the anticancer agent 3-bis(2-chloroethyl)-1-nitrosourea. The present methodology has exhibited noteworthy outcomes in augmenting the survival rates in a murine glioma model, thereby underscoring the inherent capacity of polymeric nanoparticles (NPs) to enhance the effectiveness of therapeutic interventions [102].

In line with this, in vitro investigations utilising poly(lactic-co-glycolic acid) nanoparticles (PLGA NPs) to deliver curcumin to facilitate the dissolution of amyloid-aggregates have demonstrated an absence of discernible toxicity towards the hippocampus cell culture [105]. Nevertheless, it is imperative to acknowledge that polymeric nanoparticles exhibit notable limitations, including suboptimal drug loading capacity, restricted transfection efficacy, and the propensity for particle-particle aggregation. These inherent challenges pose considerable obstacles in effectively managing and harnessing the potential of these nanocarriers [106].

Various targeting strategies have been devised to enhance the brain-specific delivery of polymer-based NPs, thereby addressing the aforementioned limitations. These approaches encompass surface modifications employing specific ligands or antibodies, as well as the utilisation of stimuli-responsive materials. In pursuit of enhancing therapeutic efficacy and mitigating untoward reactions, extensive investigations have been conducted on the utilisation of combination therapies [107].

The investigation of biodegradable polymers, namely PLGA and PCL (polycaprolactone), has been undertaken to identify potential substitutes for the quest for an optimised drug delivery system specifically tailored for cerebral applications. According to recent research findings, it has been observed that PCL nanoparticles, when loaded with curcumin, exhibit a superior ability to accumulate within the brain in comparison to PLGA nanoparticles. The findings of this study indicate that polycaprolactone (PCL) exhibits potential as a viable option for facilitating drug delivery to the central nervous system [108].

Disorders such as Huntington's disease (HD) and Alzheimer's disease (AD) are being treated with gene therapy using polymer-based nanoparticles. Small interfering RNA (siRNA) has been delivered using PLGA nanoparticles to mute the expression of particular AD-related genes [109].

Many scientific investigations have substantiated the inherent capabilities of polymer-based NPs in facilitating the transportation of pharmaceutical agents across the blood-brain barrier. These nanoparticles have exhibited promising outcomes in various domains, such as the disintegration of amyloid aggregates and the dispensation of anti-cancer therapeutics. The utilisation of polymer-based nanoparticles has been investigated in the context of glioblastoma multiforme (GBM) and brain metastases originating from breast cancer.

The aforementioned NPs exhibit inherent limitations that pose considerable challenges in their management, whether in liquid or dry states. These limitations encompass a range of factors, such as suboptimal transfection efficacy, fluctuation in performance across different batches, limited capacity for drug loading, compromised entrapment efficiency, and susceptibility to particle-particle aggregation. Moreover, it has been observed that polycation nanocarriers elicit osmotic swelling, resulting in the induction of oxidative stress, protein aggregation, impairment of mitochondrial function, and subsequent cellular demise. The activation of these NPs has the potential to induce complement system activation, thereby initiating an inflammatory response [106].

Traditional linear polymers offer restricted areas for drug loading and interaction, a significant problem [105]. It is important to remember that polymers can be of various types, such as natural polymers like albumin and chitosan, the latter of which has low toxicity levels and is biocompatible [110], although prolonged use is not recommended [111]. Additionally, Ghosh and colleagues have shown that PLGA NPs can be transported across the BBB for the treatment of gliomas while obtaining high drug solubility and passage selectivity [112].

### Dendrimer

The delivery of therapeutics across the BBB is obtained with the help of dendrimers, which are highly branched, three-dimensional polymers [113]. They are effective for storing a significant amount of drugs since they have a tightly packed periphery and a loosely packed centre that makes it easier to entrap pharmaceuticals [114]. In comparison to linear polymers, dendrimers are more advantageous due to their monodispersity, water solubility, low toxicity, high loading capacity, and abundance of changeable surface groups [115].

The surfaces of dendrimers may be changed by adding various functional groups and specific ligands, and they can be covalently coupled to pharmaceuticals or linked by electrostatic adsorption [116]. Dendrimers of several varieties, including polyamidoamine (PAMAM) dendrimers, polyhydroxylamine, and polypropylene amine, have been employed for imaging and drug delivery [117]. The polyanionic form of PAMAM dendrimers tends to be less harmful than the polycationic form. Drug transportation through the BBB can be facilitated by dendrimer modifications with ligands that target the tumour or the BBB [118].

The nanocarrier acts by five-stage mechanisms:

Stage I: Dendrimers undergo initial modifications through the introduction of surface or distance connections, thereby enhancing their biocompatibility, storage capacity, and drug release kinetics and rate.

Stage II: The primary emphasis should be placed on the BBB and its facilitation of drug delivery across the BBB. To achieve this, dendrimers are modified with specific ligands that undergo superficial modifications.

Stage III: Modified dendrimers create intricate biological connections for drug and gene therapy.

Stage IV: Dendritic nanocarriers may be incorporated with imaging agents via covalent bonds, preventing the biological distribution from being traced.

Stage V: Dendrimers release their drug payload in a controlled and sustained manner through processes such as diffusion, degradation, or stimuli-responsive release.

As a result, they offer in vivo diagnostic and imaging techniques. It should be mentioned that depending on the circumstances, the sequence and emphasis of steps II and III may vary depending on specific circumstances [113]. They have been shown to improve the uptake of medicines and DNA in the brain and have good promise for brain delivery. Lamivudine-loaded mannosylated dendrimers have shown improved antiretroviral efficacy in vitro [34].

Other targeted ligands, such as lactoferrin and transferrin, have also been effectively functionalized onto dendrimers for transcytosis over the BBB; both formulations were found to have a higher BBB crossing potential than their unmodified counterparts [111].

Nevertheless, it is imperative to acknowledge that dendrimers exhibit certain inherent limitations that warrant careful consideration. These limitations encompass batch-to-batch variability, which introduces a degree of unpredictability and necessitates meticulous monitoring and quality control measures. Additionally, the synthesis costs associated with dendrimers are notably high, demanding substantial financial resources for their production. Furthermore, the achievement of precise and targeted drug delivery using dendrimers poses significant challenges, thereby necessitating the development of innovative strategies to ensure optimal therapeutic outcomes. The potential for elevated toxicity is a notable concern in relation to cationic dendrimers, which can be ascribed to the intricate interplay between the negatively charged cellular membrane and the positively charged nanocarrier [105].

The heterogeneity of dendrimer drug-release mechanisms and short-term release kinetics are some additional dendrimer limitations that may cause the drug payload to be prematurely released prior to reaching the target location [34].

Studies on the effectiveness of dendrimers have shown that the surface can be modified for a particular purpose. For instance, PEGylation (a biochemical modification procedure of bioactive molecules with polyethylene glycol) or carbohydrate groups exclude immune response and hazardous side effects, allowing dendrimers to traverse the BBB.

### Liposomes

The spherical vesicles known as liposomes have an interior aqueous space and one or more lipid bilayers. To boost their stability in vivo, they are often composed of amphiphilic phospholipids like sphingomyelin and phosphatidylcholine [119]. The number and size of lamellae in a liposome can be used to classify them. Small unilamellar vesicles (SUVs), smaller than 100 nm in size and only contain one bilayer, are smaller than multilamellar vesicles (MLVs), which can be more than 500 nm in diameter [115].

Liposomes have been widely recognised as highly efficient delivery systems for diverse therapeutic compounds, encompassing drugs, immunisations, nucleic acids, and proteins. The surface of these entities exhibits a remarkable propensity for facile modification, rendering them highly amenable to customization. Moreover, their inherent biocompatibility endows them with the ability to seamlessly integrate into biological systems. Notably, these entities possess an amphiphilic nature, allowing them to interact with both hydrophilic and hydrophobic environments. Additionally, their extended circulation times further enhance their utility and potential for various applications. Surface modification techniques play a crucial role in tailoring the properties and objectives of liposomes. These techniques encompass the incorporation of various entities, including polymers, polysaccharides, peptides, or antibodies, to achieve the desired specifications. PEGylation is a widely employed strategy in biomedical research aimed at improving the biodistribution of liposomes and mitigating their immune clearance [36]. Moreover, the utilisation of transferrin or apoE has been demonstrated as a viable approach for the fabrication of liposomes, thereby facilitating the delivery of gene therapy and medications across the BBB [120].

Another approach to enabling liposomes to penetrate the BBB via receptor-mediated transcytosis (RMT) is to combine them to target ligands that link to receptors on the surface of brain endothelial cells [115].

Additional strategies encompass the modification of liposomal surfaces by integrating stealthy polymers or other compounds, thereby attenuating the immunological response and prolonging their circulation time within the vascular system. Pharmaceutical compounds that exhibit responsive behaviour in the presence of specific stimuli, such as alterations in pH or temperature, have been investigated for their potential encapsulation within liposomal structures. This encapsulation strategy enables the controlled and triggered release of the therapeutic agent at the desired anatomical site [113].

The reticuloendothelial system eliminates typical liposomes, which results in a shorter circulation period. Transferrin, glucose, and TAT peptide have all been investigated for potential surface modifications to improve the dispersion of loaded liposomes in the brain. However, there is concern regarding the non-specific delivery of highly active drugs to healthy, non-cancerous brain tissues across the BBB [34].

Dual-targeting liposomal formulations, where liposomes are coupled with both transferrin and folate, have been created to solve this problem. This study shows the effectiveness of liposomes in their ease of surface modification [121].

SLNs are a class of nanocarriers that exhibit similarities to liposomes yet possess distinct structural characteristics. Unlike liposomes, SLNs consist of solid lipid spheres, wherein the lipid matrix is predominantly lipophilic rather than organised into a lipid bilayer. The stability of solid lipid nanoparticles (SLNs) surpasses that of liposomes, rendering them a more favourable option in drug delivery systems. Additionally, the manufacturing process of SLNs is characterised by enhanced efficiency, allowing for rapid production compared to liposomes. Moreover, SLNs exhibit superior efficacy in facilitating the transportation of drugs, thereby enhancing their therapeutic potential. Extensive research has been conducted on the utilisation of liposomes and SLNs as potential strategies for traversing the BBB in the context of brain tumour treatment [122, 123].

An approved drug called liposomal doxorubicin is used to treat a number of neoplasms, including brain tumours [124]. It is possible that FUS will produce better liposomes that deliver medications., such as cisplatin or doxorubicin, to increase BBB permeability and decrease tumour growth in mice. FUS enhances the relative permeability of the BBB in a time-dependent manner: an experimental study" by Yufeng Zhou *et al.* [125]. Rat glioma tumours can be targeted with paclitaxel-carrying liposomes that can pass through the BBB. It is possible to enhance the ability of drug-loaded liposomes to target gliomas and increase animal models' lifespans by conjugating them with particular vectors [105].

### Carbon nanotubes

Due to their special characteristics, carbon nanotubes (CNT) are promising candidates for medication delivery in the CNS. The flexibility of the carrier can be influenced by the number of graphene layers, with fewer layers allowing for more flexibility [126]. Multi-walled carbon nanotubes functionalized with an amine group (MWCNTs-NH_3_^+^) have been shown to successfully pass the BBB by transcytosis in both in vitro and in vivo rodent models [127]. These findings imply that functionalized carbon nanotubes may represent a therapeutic alternative for CNS conditions that call for effective medication transport to the brain, such as brain tumours [128].

With some chemical substances, CNTs can easily undergo surface functionalization, which may explain some of the variance in their physical and biological characteristics. However, there are drawbacks, including toxicity, batch-to-batch fluctuation, and high production costs [111].

In a study, MWCNTs, which, to improve their bioavailability, were additionally surface modified with phospholipids and polysorbate., were exposed to Berberine, an anti-Alzheimer's medication. SHSY-5Y cells, a triple-subcloned cell line generated from the SK-N-SH neuroblastoma cell line, performed in vitro experiments, and the results showed no detectable toxicity. Rats' memory improved in vivo tests after the nanoformulation was given to them. Rat brain and plasma tissues displayed drug absorption, demonstrating BBB crossing by the nanoformulation [129]. Consequently, drug-loaded phospholipid/polysorbate-coated MWCNTs may aid AD patients in reducing amyloid accumulation at the target region.

These nanostructures' permeability to the brain decreased as the temperature increased, demonstrating the energy-dependent mechanism of these drug delivery systems. However, significant absorption by astrocytes and considerable accumulation of amino-functioned single-walled CNTs in brain tissue were noted, suggesting these CNTs could serve as CNS drug delivery vehicles [127]. To comprehend their processses of action and improve their characteristics for therapeutic application, more study is necessary [130].

Conclusively, the polymeric nanoparticles hold great promise in drug administration due to their core-shell structure and hydrophilic polymer coating. However, challenges such as suboptimal drug loading capacity and particle aggregation necessitate further optimization. Strategies like surface modifications with ligands and combination therapies are explored to enhance brain-specific drug delivery and efficacy, particularly in diseases like Alzheimer's and glioblastoma.

Similarly, the highly branched polymers' dendrimers exhibit advantages such as high loading capacity and precise surface modifications. Their potential in drug delivery across the BBB is acknowledged, but concerns about batch variability and potential toxicity underline the need for careful consideration.

Liposomes, spherical vesicles with lipid bilayers, offer efficient drug delivery options with inherent biocompatibility. Surface modifications through various compounds allow targeted delivery and controlled release of therapeutic agents. Solid lipid nanoparticles (SLNs), resembling liposomes but with a more stable structure, are also explored for drug delivery to treat brain tumours.

CNTs, with their unique characteristics, show promise in CNS drug delivery. Functionalized CNTs can effectively cross the BBB and may serve as carriers for various therapeutic substances. However, challenges related to toxicity and production costs need to be addressed. Surface-modified MWCNTs have the potential to deliver drugs across the BBS. However, their brain permeability decreased with higher temperatures, indicating an energy-dependent mechanism. Notably, they were absorbed significantly by astrocytes, suggesting their potential as CNS drug delivery vehicles, but further research is needed to optimize their therapeutic application.

## Various models to study BBB

Finding innovative methods to administer medications to the brain has benefited greatly from using BBB in vitro models [131]. By using these models, researchers have been able to test the effectiveness of nanomaterials as drug delivery agents to cross the blood-brain barrier. Various NMs, such as liposomes, dendrimers, and nanoparticles, have demonstrated efficacy in enhancing drug transport across the BBB. These NMs can be customized to transport diverse types of substances and can enhance the efficacy of drugs by increasing their specificity, reducing toxicity, and facilitating their delivery to specific areas of the brain [132,133].

The capacity of functionalized NMs to preferentially attach to receptors on the surface of BBB endothelial cells, which increases their ability to pass the barrier, is a significant benefit. Understanding these transport systems and determining the safety of nanomaterials for delivering medications to the brain have benefited greatly from in vitro research [134].

Overall, in vitro models of the BBB have played an important role in advancing drug delivery strategies for overcoming the BBB [135]. NMs have proven to be effective in enhancing drug delivery to the brain, and in vitro research has provided valuable information about their mechanisms of transport and safety. Additionally, in vitro models of the BBB can be used for drug screening, including the use of microfluidic devices for high-throughput screening [136].

Two popular in vitro models for investigating the BBB and assessing the feasibility of NMs in crossing this barrier for drug delivery to the brain are cell-based models and microfluidic devices.

Cell-based models are frequently utilized in vitro models to study the BBB. These models involve using various cells to imitate the BBB structure. For instance, a widely used cell-based model entails cultivating BMECs on a transwell membrane to form a monolayer with tight junctions, mimicking the BBB structure [137,138]. A more intricate in vitro BBB model can be formed by including other cell types like astrocytes and pericytes, along with brain microvascular endothelial cells (BMECs), to mimic the BBB structure [2]. Cell-based models offer a controlled and reproducible experimental system for drug screening and studying BBB transport mechanisms, which is a significant advantage [139]. Although cell-based models are valuable tools for studying the BBB, they have some limitations. For instance, they lack fluid shear stress and other components of the BBB, which can result in inaccurate estimations of drug transport [140].

In vitro BBB models offer a reliable and standardized experimental platform for drug screening and for studying the mechanisms of BBB transport [138], but it is important to acknowledge that these models have limitations, including the absence of important BBB components and fluid shear stress, which can lead to overestimation or underestimation of drug transport [140]. Cell-based models used to study the BBB in vitro, referred to as static models, offer several benefits, including reproducibility, scalability, and cost-effectiveness. However, these models are limited in their ability to mimic the physiological environment of the BBB, lacking fluid shear stress and other important components [139]. Nonetheless, they fall short in simulating the dynamic fluid flow and mechanical forces present in vivo, which can significantly influence drug transport and cell behaviour [140].

Other static models involving different types of cells, including astrocytes and pericytes, can be used in addition to the commonly used transwell model with BMECs to create more complex in vitro BBB models for studying drug transport and evaluating the potential of nanomaterials for overcoming the BBB.

Static in vitro BBB models relate to using cultured cells in a static environment to simulate the BBB, known as static in vitro BBB models. These models are beneficial in investigating drug efficacy, drug transport, and BBB permeability in vitro. The BBB consists of three key cell types: brain endothelial cells, astrocytes, and pericytes [141]. Static in vitro models usually involve culturing brain endothelial cells on a permeable membrane with astrocytes and pericytes in the culture medium. However, simpler models that only consist of endothelial cells can also be employed. [142].

Static in vitro models are advantageous due to their ability to provide a reproducible, scalable, and cost-effective experimental system for studying BBB permeability, drug transport, and drug efficacy *in vitro* [143]. Static in vitro models offer the advantage of being less complex than in vivo models, making interpreting results easier [144]. One such limitation of this model is the absence of dynamic fluid flow and mechanical forces that exist in vivo and can impact cell behaviour and drug transport [145].

Numerous studies have employed static in vitro models to explore the capacity of NMs in crossing the BBB. For instance, Yan *et al.* [146] conducted research using a static in vitro BBB model to examine the uptake and transportation of curcumin-containing nanoparticles by brain endothelial cells. The findings suggested that the NMs could traverse the BBB and gather in the brain tissue, indicating their potential as an efficient drug delivery system for treating neurological disorders.

Microfluidic devices provide a more realistic in vitro BBB modelling platform by incorporating various cell types and emulating the fluid dynamics of the brain. These devices enable the simultaneous culture of BMECs with other cells, such as astrocytes and neurons, in a precisely controlled microenvironment that closely resembles the structure and function of the BBB [147]. Microfluidic devices have been employed to investigate the permeability of nanoparticles across the BBB, assess the toxicity of NMs on BBB endothelial cells, and screen promising drugs for their ability to cross the BBB [148]. To investigate the potential of nanomaterials for drug delivery to the brain, various models have been utilized, including cell-based models and microfluidic devices. These models provide a means to comprehend the mechanism of nanoparticle transport, assess the safety of nanomaterials, and screen for potential drug candidates.

A study by T. D. Brown *et al.* [149] utilized a microfluidic device to examine the ability of nanocarriers to cross the BBB. The microfluidic device consisted of two compartments separated by a permeable membrane coated with astrocytes to replicate the BBB structure. One side of the membrane was cultured with brain endothelial cells, and NMs were introduced to the other. The study found that the transport efficiency of nanoparticles across the BBB was affected by their size and surface charge, and the presence of astrocytes improved their transport efficacy. This article highlights the potential of microfluidic devices as a valuable tool for understanding the mechanism of nanoparticle transport across the BBB and developing effective drug delivery [150] strategies for the treatment of neurological disorders [149].

One example of a dynamic in vitro model is a microfluidic-on-a-chip system. This type of laboratory model is made by designing tiny channels on a microchip [147]. These models have many benefits, like being more like real life and helping us learn more about the BBB and how to deliver drugs to the brain. They can mimic complex shapes and movements in the brain and can be used to test many different drugs simultaneously [151]. Overall, dynamic in vitro models of the BBB, like microfluidic-on-a-chip systems, are very important for understanding how the brain works and how we can treat diseases that affect the brain.

A study by W. Zhang *et al.* [152] used a microfluidic BBB model to evaluate the transport of gold nanoclusters (AuNCs) across the BBB for potential drug delivery applications. The study used a microfluidic BBB model with human brain microvascular endothelial cells (HBMECs) and astrocytes to mimic the BBB and NVU. AuNCs were synthesized and loaded with the anti-cancer drug doxorubicin and introduced to the BBB model.

The study found that the AuNCs were able to cross the BBB model and were taken up by the surrounding astrocytes, indicating their potential as drug-delivery agents. The study demonstrates the potential of dynamic in vitro models of the BBB, such as microfluidic BBB models, to enhance our comprehension of nanomaterial transport across the BBB and create efficient drug delivery systems for the treatment of neurological diseases [152].

## Safety concerns of nanoparticles for delivery across BBB

NMs offer a promising avenue for delivering drugs to the brain by overcoming the BBB [153]. However, it is essential to address safety concerns associated with the use of nanomaterials for drug delivery. In this topic, we will examine the safety considerations of nanomaterials as drug carriers for crossing the BBB, emphasizing the importance of evaluating and minimizing potential harm while maximizing therapeutic benefits.

### Physicochemical properties and potential toxicity

NMs possess unique physicochemical properties that make them promising candidates for drug delivery to the brain. Manipulating their size, shape, composition, and surface charge can enhance their ability to penetrate the BBB. However, it is crucial to carefully evaluate the potential toxicity of these NMs to ensure their safe application [154]. Understanding the relationships between physicochemical properties and toxicity will facilitate the development of effective and safe nanomaterial-based drug delivery systems for the brain.

**Size-dependent**: The influence of nanoparticle size on their transport across the BBB and potential toxicity is significant. It is believed that surface-coated NMs, resembling LDL (low-density lipoprotein), utilize receptor-mediated mechanisms to cross the BBB [155]. Polysorbate 80 acts as a link between NMs and apolipoproteins, particularly ApoE (apolipoprotein E), promoting LDL receptor-mediated transcytosis [156]. NMs with sizes similar to LDL (around 20-25 nm) have shown optimal effectiveness in drug delivery across the BBB. Smaller NMs tend to degrade more rapidly within endothelial cells, leading to a faster drug release into the brain. Thus, nanoparticle size is crucial in drug delivery to the brain [157].

**Surface charge**: In addition to the physical properties of endothelial cell membranes and the size of NMs, the surface charge of nanomaterials plays a significant role in their transport across the BBB. Electrostatic, specifically cationic, can interact with anionic areas on the BBB endothelium. This interaction may increase endothelial cell permeability by potentially disrupting the junctions between cells [158]. In vitro studies have shown that cationic nanoparticles have higher brain translocation compared to anionic or neutral NMs [159]. The size and charge of colloidal drug carriers are essential in determining their ability to deliver drugs or nanoparticles across the BBB or into the brain parenchyma [160]. However, a limited amount of in vivo data is available regarding the brain permeability of cationic NMs.

In a study by Koziara *et al.* [161], it was shown that administering negatively charged NMs to animals at doses between 100 and 200 mg per animal caused their accumulation in the cellular matrix without causing any apparent neurotoxic consequences. However, as demonstrated in the study by Koziara *et al.* [161], the amount of BBB opening in the presence of anionic nanoparticles is only proportional to the free fraction of nanoparticles in formulations [162]. Therefore, further investigations are required to determine the optimal concentration of negatively charged BMs that can be achieved in the brain without causing any noticeable neurotoxicity.

**Shape-related toxicity**: Nanomaterials with a high aspect ratio, such as nanorods or nanowires, have been found to exhibit improved penetration across the BBB compared to spherical NMs. Their elongated shape enhances interaction with BBB endothelial cells, facilitating translocation through tight junctions or cellular uptake mechanisms. Similarly, needle-like or fibre-like NMs have demonstrated superior penetration capabilities attributed to their shape-induced physical disruption of the BBB [163]. Moreover, the shape of NMs influences cellular uptake mechanisms, as elongated structures like nanotubes or nanofibers can be internalized through endocytosis or receptor-mediated pathways, enhancing their transport across the BBB. Additionally, specific nanomaterial geometries, such as nanoneedles or nanowires, can exert mechanical forces on tight junctions, temporarily disrupting them and enabling nanomaterial passage. It is also important to consider stability and aggregation since nanomaterial shape affects their propensity for aggregation, potentially hindering BBB crossing [164]. Ensuring the stability of nanomaterials under physiological conditions is crucial for maintaining their shape-related properties during BBB transport.

**Chemical composition**: The chemical composition of nanomaterials is a critical factor in determining their properties and potential toxicity when used for drug delivery across the BBB. Liposomes, composed of lipid bilayers and polymeric NMs made of biocompatible polymers, are commonly utilized for their versatility in encapsulating and delivering drugs to the BBB [165]. Dendrimers, highly branched macromolecules, offer tailored surface chemistry for improved drug stability and targeting, but their potential toxicity requires careful consideration [166]. Carbon-based NMs like graphene and carbon nanotubes possess unique properties that make them attractive for BBB drug delivery, but thorough evaluation is necessary to ensure their safety and effectiveness [167].

**Cellular toxicity**: Ensuring the safe application of NMs in BBB drug delivery requires a thorough assessment of their potential cellular toxicity. Understanding how NMs interact with different brain cell types is vital for developing effective strategies that minimize adverse effects [105]. By comprehensively evaluating the advantages and risks associated with NMs-induced cellular toxicity, researchers can pave the way for developing safer and more efficient drug delivery systems [168]. These systems have the potential to overcome the BBB, providing targeted treatments for various neurological disorders while minimizing harm to the cells of the brain.

**Neurons**: Assessing the potential toxicity of nanomaterials on neurons is of utmost importance to ensure their safe application in BBB drug delivery. By understanding their impact on neuronal viability, morphology, and functionality, researchers can develop effective strategies with targeted drug delivery to specific neuronal populations and potential neuroprotective effects [169]. In a study investigating the impact of metal-based NMs on neurons, researchers exposed primary neuronal cultures to silver NMs (AgNPs) and gold NMs (AuNPs). They found that the NMs caused a dose-dependent decrease in neuronal viability, indicating potential toxicity. Additionally, the NMs disrupted neuronal processes and impaired synaptic activity, as evidenced by altered calcium influx and reduced expression of synaptic proteins [170].

**Astrocytes**: Astrocytes play a crucial role in maintaining brain homeostasis and supporting neuronal function [171]. NMs can influence astrocyte viability, activation, and the release of inflammatory mediators. In a study examining the effects of carbon nanotubes (CNTs) on astrocytes, researchers exposed cultured astrocytes to different concentrations of CNTs. They observed that CNT exposure led to a dose-dependent decrease in astrocyte viability, indicating potential toxicity. Additionally, the CNTs triggered the activation of astrocytes, as evidenced by an increase in the expression of activation markers such as GFAP (glial fibrillary acidic protein). Furthermore, Interleukin-6 (IL-6) and tumour necrosis factor-alpha (TNF-) were released as a result of the exposure to CNTs, indicating an inflammatory reaction. This study underscores the importance of evaluating the impact of NMs on astrocytes and their potential role in neuroinflammation, which can have implications for BBB in drug delivery strategies [172].

**Microglia**: Inflammation and immunological responses are greatly influenced by microglia, the central nervous system's resident immune cells. The production of reactive species, pro-inflammatory cytokines, and an inflammatory response can all be caused by NMs activating microglia. Evaluating microglial activation, cytokine release, and oxidative stress markers provides insights into the potential toxicity of nanomaterials on microglia [173]. In order to examine how silver NMs (AgNPs) affect microglia, researchers subjected cultured microglial cells to various AgNP concentrations. AgNPs induced oxidative stress in microglia, as indicated by elevated levels of reactive oxygen species (ROS). These findings highlight the potential for nanomaterial-induced inflammatory responses and oxidative stress in microglia, underscoring the importance of assessing the potential toxicity of nanomaterials on microglia in the context of blood-brain barrier drug delivery [174].

**Endothelial cells (ECs)**: BBB endothelial cells form a crucial barrier and regulate the transport of molecules into the brain. NMs can interact with BBB endothelial cells, affecting their viability, integrity, and tight junction proteins [142]. Disruption of tight junctions can compromise the integrity of the BBB. Evaluating EC viability, barrier integrity, tight junction proteins, and inflammatory responses is important in assessing the potential toxicity of NMs on BBB endothelial cells. In a study investigating the effects of liposomes on BBB endothelial cells, researchers exposed cultured endothelial cells to liposomes of different compositions. They found that exposure to liposomes led to changes in endothelial cell viability and morphology, suggesting potential toxicity. Additionally, the liposomes affected the integrity of the tight junction proteins that regulate the permeability of the BBB, indicating potential disruption of the barrier function [142].

**Systemic toxicity**: To date, systemic toxicity has been a significant challenge in the field of drug delivery to the brain, with systemic administration being the most extensively studied approach. To overcome this obstacle, it is crucial to develop a design where the active drug can be encapsulated within non-toxic NMs and capable of crossing the BBB [13]. Additionally, various approaches have been developed to enhance BBB permeability, such as the injection of hyperosmolar mannitol to induce reversible disruption [32] or the application of ultrasound as a physical stimulus [12,175]. However, BBB disruption can result in the influx of neurotoxic substances, leading to substantial brain damage [13]. Thus, in order to treat neurological diseases while avoiding neuronal damage brought on by BBB rupture, innovative drug modification tactics might be extremely important. Lipid-based NMs have demonstrated potential as a secure and efficient way to penetrate the BBB [176].

After administering NMs for BBB drug delivery, it is important to consider their potential systemic toxicity. When NMs are administered, they can undergo various processes such as absorption, distribution, metabolism, and excretion (ADME) within the body. Although NMs offer promising benefits, it is necessary to assess their adverse effects on organs and tissues beyond the targeted site. The biodistribution and accumulation of NMs can be influenced by size, surface properties, and targeting ligands, which influence the distribution and accumulation of nanomaterials in various body areas. Accumulation in unintended organs, such as the liver, spleen, and lungs, raises concerns about off-target effects. Additionally, specific types of NMs, like carbon-based ones [177], have shown potential toxicity in the lungs, immune system, and liver. Since the brain is the intended target, evaluating neurotoxicity is crucial. This includes assessing potential neuronal cell death and disruptions in neurodevelopment caused by nanomaterial exposure [178].

Intranasal administration has become a potential and non-invasive alternative to conventional parenteral methods for medication delivery to the brain. This form of administration has a number of benefits, including preventing hepatic metabolism, lowering drug buildup in non-target organs, and decreasing systemic adverse effects. Due to its early start of the effect, quick absorption, non-invasiveness, absence of tissue damage, and convenience of usage, intranasal medication delivery is often employed [179,180].

In a study conducted by Choi *et al.* [181], the biodistribution of polymeric NMs was investigated in mice by intravenous injection. The findings revealed that the NMs predominantly accumulated in the liver and spleen, suggesting the possibility of off-target effects in these organs. The study highlighted the importance of understanding the distribution patterns of NMs within the body to assess their potential impact on non-targeted organs. This information is crucial in the development of drug delivery systems to minimize off-target effects and maximize therapeutic efficacy [181].

J. Duan *et al.* [182] conducted a study in 2021 to investigate the potential neurotoxicity of silica NMs using zebrafish models. The study aimed to assess the effects of exposure to silica NMs on neuronal cells and neurodevelopment. The investigations revealed that exposure to silica NMs resulted in neuronal cell death and disrupted normal neurodevelopment processes. These findings emphasize the importance of conducting thorough evaluations and assessments of nanomaterials for drug delivery to the brain. It highlights the need for careful consideration of potential neurotoxic effects when designing and selecting NMs for brain drug delivery strategies to ensure the safety and efficacy of therapeutic interventions [182].

### Safety measures for nanomaterials in drug delivery to BBB

The potential of NMs as drug delivery systems to penetrate the BBB and treat neurological disorders is immense. However, ensuring the safety of these NMs is crucial for their effective and safe utilization. Surface modification and rational design strategies significantly enhance the safety profile of NMs in BBB drug delivery [168].

This will focus on the importance of safety measures, particularly rational design and surface modification, in enhancing the safety and efficacy of NMs for crossing the BBB. It will explore specific techniques such as PEGylation, coating NMs with biocompatible polymers, and ligand targeting, highlighting their potential to minimize nanomaterial toxicity [183]. Additionally, the review covers preclinical safety evaluation methods and regulatory considerations for NMs in brain drug delivery, providing a comprehensive understanding of the safety aspects associated with using NMs for BBB drug delivery systems.

PEGylation is a widely adopted technique in nanomedicine, involving the attachment of polyethylene glycol (PEG) chains to nanomaterial surfaces, which creates a hydrophilic shield around the nanomaterial, mitigating recognition and clearance by the immune system. This reduction in immune response enhances biocompatibility and minimizes the risk of adverse reactions. PEGylation also contributes to diminished interactions between NMs and biological components, reducing cellular uptake and potential toxicity. The steric hindrance provided by PEG chains prevents nanomaterial aggregation, ensuring improved stability in biological environments. Moreover, PEGylation extends the circulation time of NMs in the bloodstream, facilitating prolonged drug delivery to the brain. Additionally, surface coating with biocompatible polymers represents another strategy to enhance the safety of NMs in BBB drug delivery [168,184,185].

**Polyethyleneimine (PEI)**: Coating NMs with PEI imparts a positive surface charge, enhancing cellular uptake and transport across the BBB. PEI coatings also protect NMs from degradation and reduce their potential toxicity [186,187].

**Chitosan**: Chitosan, a natural polysaccharide, is frequently used for surface modification of NMs. It forms a protective layer, improves stability, and reduces interactions with biological components, thereby enhancing biocompatibility and minimizing cytotoxicity [183].

**Ligand targeting**: Ligand targeting involves attaching specific ligands to the surface of NMs to facilitate their interaction with receptors or transporters in the BBB. This surface modification technique enables targeted drug delivery and reduces off-target effects [188].

**Targeting BBB receptors/transporters**: Conjugating ligands that can bind to receptors or transporters expressed in the BBB allows NMs to selectively interact with these specific sites, facilitating their transport across the barrier and enhancing drug delivery to the brain [183].

**Enhanced efficiency and reduced systemic toxicity**: Ligand-mediated targeting improves drug delivery efficiency by increasing therapeutics accumulation at the desired site while minimizing their distribution to non-target tissues. This approach reduces systemic toxicity and improves the therapeutic index [183].

Ensuring the safety of nanomaterials intended for brain drug delivery is critical to their development. Comprehensive safety evaluations involve a multifaceted approach, encompassing both in vitro assays and animal models, alongside adherence to regulatory considerations. In vitro assays play a pivotal role, encompassing tests for cytotoxicity, cellular uptake, reactive oxygen species (ROS) generation, and blood-brain barrier (BBB) permeability. Researchers also delve into how nanomaterial design and surface modifications impact these in vitro safety assessments [189].

Animal models, particularly rodents such as mice and rats models, enable the assessment of acute and chronic toxicities, distribution patterns, and long-term effects. Neurobehavioral assessments, histopathological analyses, and biodistribution studies collectively contribute to a thorough understanding of the safety profile of NMs [168,190].

**Importance of long-term effects, immunotoxicity and genotoxicity**: Regulatory authorities emphasize the assessment of long-term effects, immunotoxicity [188], and genotoxicity during the safety evaluation of NMs for brain drug delivery. Understanding the potential risks associated with prolonged exposure, immune responses, and DNA damage is crucial for obtaining regulatory approval. Comprehensive evaluations are necessary to ensure the safe translation of nanomaterial-based drug delivery systems for brain disorders [191].

**Regulatory guidance and reviews**: Several regulatory guidelines provide comprehensive frameworks for evaluating the safety of nanomaterials. The International Council for Harmonisation (ICH) guidelines, the U.S. Food and Drug Administration (FDA) nanotechnology initiatives [192] and the European Medicines Agency (EMA) guidelines offer valuable guidance for nanomaterial safety assessments. Additionally, reviews and studies focus on safety evaluation methodologies, contributing valuable insights into regulatory considerations [193].

By implementing rational design and surface modification strategies, the safety profile of nanomaterials for BBB drug delivery can be improved. Approaches such as PEGylation, surface coating with biocompatible polymers, and ligand targeting show promise in minimizing nanomaterial toxicity. In vitro assays and animal models serve as important tools for safety evaluation [192], while regulatory guidelines emphasize the assessment of long-term effects, immunotoxicity, and genotoxicity. A comprehensive understanding of these safety measures is essential for the successful translation of nanomaterial-based drug delivery systems for brain disorders.

In summary, the rational design and surface modification strategies significantly enhance the safety profile of nanomaterials intended for BBB drug delivery. Key approaches such as PEGylation, surface coating with biocompatible polymers, and ligand targeting have shown promise in minimizing nanomaterial toxicity and improving their efficacy. PEGylation, a widely adopted technique, involves attaching polyethylene glycol (PEG) chains to nanomaterial surfaces, creating a hydrophilic shield to reduce immune recognition and clearance. This approach enhances biocompatibility, minimizes potential toxicity, and extends stability and circulation time, ultimately enabling prolonged drug delivery to the brain. Coating nanomaterials with biocompatible polymers like polyethyleneimine (PEI) and chitosan provides a positive surface charge and forms a protective layer, enhancing stability and biocompatibility while reducing cytotoxicity. Ligand targeting, achieved by attaching specific ligands to nanomaterial surfaces, enables interaction with BBB receptors or transporters, facilitating targeted drug delivery and reducing off-target effects.

In terms of safety evaluation, in vitro assays and animal models are crucial tools. In vitro assays assess cytotoxicity, cellular uptake, reactive oxygen species (ROS) generation, and BBB permeability. Animal models, particularly rodents, provide valuable insights into acute and chronic toxicities, distribution patterns, and long-term effects of nanomaterials. Evaluating long-term effects, immunotoxicity, and genotoxicity is crucial for regulatory approval, aligning with international guidelines such as those from the International Council for Harmonisation (ICH), U.S. Food and Drug Administration (FDA), and European Medicines Agency (EMA).

In essence, a comprehensive understanding of safety measures, encompassing rational design, surface modification, in vitro evaluations, animal studies, and regulatory considerations, is imperative for the successful and safe translation of nanomaterial-based drug delivery systems for treating brain disorders. These safety measures are essential to advance nanomaterial-based therapies and enhance their potential to revolutionize the treatment of neurological conditions while ensuring patient safety and well-being.

## Applications of nanomaterials in delivering drugs across BBB

Nanomaterials, such as micelles and liposomes, have emerged as promising tools in cancer therapy, offering several advantages for the delivery of chemotherapeutic agents. These nanocarriers possess a hydrophobic core and a hydrophilic shell, enabling them to solubilize insoluble drugs. By modifying the surface of nanocarriers, such as through PEGylation, they can passively target tumours and inflamed tissues by exploiting fenestrated vasculature, resulting in higher drug concentrations at the tumor site [194]. Notably, clinical studies for a number of polymeric micelles carrying anticancer medications, such as NK012, NK105, NK911, NC-6004, and SP1049C, are now being conducted [195].

One successful example is Genexol-PM, a paclitaxel-loaded polymeric micelle formulation approved for breast cancer treatment [196]. Another class of NMs, dendrimers, has demonstrated great potential in localizing, imaging and delivering anticancer drugs [197]. These highly branched macromolecules can be functionalized with various ligands for targeted drug delivery [198]. For instance, a polyfunctional dendrimer system with folic acid as the targeting ligand, fluorescein for imaging, and methotrexate as the anticancer drug have shown successful results in vitro [197].

Carbon nanotubes offer unique advantages in cancer treatment with their large surface area and capability to load hydrophobic drugs. They can easily encapsulate water-insoluble drugs within their hydrophobic hollow interior. Moreover, the outer surface of carbon nanotubes can be functionalized to specifically target cancer receptors or serve as contrast agents for imaging purposes [199]. Similarly, buckminsterfullerene C60, a spherical molecule, and its derivatives are being evaluated for their potential in cancer therapy [200].

In the field of diagnosis, quantum dots present significant advantages for monitoring various biological events simultaneously. These fluorescent nanoparticles can be excited using white light and conjugated with biomolecules, allowing for long-term tracking and probing of different bio-mechanisms. This technology enables tagging multiple biological molecules with nanodots of specific colours [201].

Theranostic NMs, which combine medication and an imaging agent in a single formulation, have also attracted much attention. Drug conjugates, dendrimers, micelles, vesicles, core-shell particles, and carbon nanotubes are just a few examples of the various types of NMs that have been investigated for tracking nanoparticle routes, locating at the target location, and evaluating therapeutic response [202].

Nanotechnology has also made significant contributions to the treatment of HIV and AIDS. Polymeric NMs have been developed to deliver antiretroviral (ARV) drugs intracellularly, including to the brain, enhancing the effectiveness of treatment. These NMs can overcome barriers such as the mucosal epithelial barrier and the BBB, enabling successful delivery of anti-HIV medications. Combining nanotechnology with vaccinations offers a potential strategy to prevent HIV infections [203].

In nutraceutical delivery, nanotechnology has played a vital role in improving the bioavailability and efficacy of orally consumed nutraceuticals. Nanoparticle formulations have been investigated to enhance the dissolution mechanisms of lipophilic nutraceuticals, such as fat-soluble vitamins (A, D, E, and K) and polyunsaturated lipids. These formulations improve the effectiveness of nutraceuticals and provide additional health benefits, reducing the risk of chronic illnesses [204, 205].

## Future prospects of nanomaterials

The field of nanomedicine is currently a highly captivating area of research, offering promising prospects for disease diagnosis and combined diagnosis-therapy approaches. Several key aspects require thorough investigation and development. Firstly, detailed tracing and analysis of nano-based drug delivery systems (NDDSs) are necessary to understand their integrity, surface characteristics, pharmacokinetics, biodistribution, and immunological effects [206-208]. Advanced technologies and methods will be crucial for addressing these challenging aspects. Secondly, establishing a normative evaluation framework and using rational animal models are essential to assess the efficiency of NDDSs [209]. Target specificity, tissue exposure, safety profiles, patient considerations, and commercial potential should guide the evaluation process [210]. Effective collaboration between biologists, mathematicians, chemists, and medical scientists is crucial in the design of nanomedicine-based drug delivery systems (NDDSs) that offer clinical value. Furthermore, priority should be given to the development of NDDSs that are structurally simple and reproducible, as they hold immense potential for widespread patient access. Although there is a need for further advancement in regulatory mechanisms and safety assessments for nanomedicines, it is important to recognize that nanomedicine has already brought a transformative shift in drug discovery and administration within biological systems.

By leveraging stimuli-responsive materials and advanced nanotechnology, these systems offer precise control over drug release, improving therapeutic efficacy while minimizing off-target effects [211]. Non-invasive delivery methods such as intranasal delivery, focused ultrasound, and magnetic targeting are being explored to overcome the BBB without invasive procedures, enhancing drug delivery efficiency and reducing associated risks. Combination therapies utilizing NMs present an opportunity to synergistically target multiple pathways in neurological disorders, leading to improved treatment outcomes [212]. Personalized medicine approaches tailored to individual patient characteristics, including genetic profiles and disease subtypes, can optimize treatment effectiveness. Regulatory considerations need to be addressed to adapt to the unique challenges of nanomaterial-based drug delivery systems. This involves developing standardized evaluation protocols to ensure patient safety and facilitate regulatory approval [213]. Future research will prioritize the development of scalable manufacturing techniques for nanomaterials utilized in drug delivery applications [214]. The primary focus will involve enhancing the synthesis and fabrication methods to achieve reproducibility, cost-effectiveness, and the ability to produce on a large scale. Several examples of scalable manufacturing processes include continuous flow synthesis, spray drying, and microfluidics. These methods offer efficient and high-throughput production of NMs, allowing for precise control over particle size, shape, and surface properties [215]. The advantages of scalable manufacturing processes in this context are numerous. Large-scale production facilitates broader availability of drug delivery systems based on NMs, increasing patient access. Moreover, such processes can substantially reduce production costs, making nanomedicine-based therapies more affordable and accessible. Improved reproducibility ensures consistent quality and performance of the NMs, thereby enhancing the reliability of drug delivery systems. Nonetheless, there are certain drawbacks to consider. Scaling up the production process may introduce challenges in preserving the stability and integrity of NMs [216]. It becomes increasingly crucial to ensure batch-to-batch consistency as the production volume expands. Additionally, complex manufacturing processes may require sophisticated equipment and specialized expertise, leading to additional initial setup and operational expenses. Regulatory compliance and quality control have also become essential in large-scale production to ensure the safety and efficacy of the resulting products [217].

As the production of nanomaterial-based drug delivery systems expands, the implementation of robust quality control measures and advanced characterization techniques becomes vital to assess their integrity, safety, and performance [218]. Establishing standardized protocols and guidelines for quality control testing, encompassing physical and chemical characterization, particle size analysis, surface charge determination, and drug loading efficiency, is essential. These measures ensure consistency, meet regulatory requirements, and provide valuable insights. The advantages of implementing quality control and characterization processes are manifold. Rigorous quality control guarantees the reliability, safety, and efficacy of nanomaterial-based drug delivery systems. Standardized protocols enable comparison and evaluation of different production batches, ensuring consistency and quality assurance [219]. Advanced characterization techniques provide a comprehensive understanding of the NMs' physicochemical properties and performance, facilitating optimization and further development. Nevertheless, there are certain disadvantages to consider [220]. The implementation of comprehensive quality control measures introduces complexity and additional costs to the production process. Advanced characterization techniques may require specialized equipment, expertise, and time, potentially impacting production timelines and expenses.

The large-scale production of nanomaterial-based drug delivery systems necessitates strict adherence to regulatory guidelines and safety assessments to ensure patient safety and facilitate regulatory approval. Regulatory agencies are continuously updating and refining their guidelines for the regulation of nanomedicine products [221]. Prospects involve harmonizing regulatory requirements to establish clear pathways for the approval and commercialization of nanomaterial-based drug delivery systems [222]. Adhering to regulatory guidelines offers several advantages. It ensures the safety, quality, and efficacy of nanomaterial-based drug delivery systems. Regulatory approval provides a framework for market access and clinical translation, increasing the potential for widespread adoption of these therapies. However, there are certain disadvantages to consider [210]. Meeting regulatory requirements can be time-consuming and costly, particularly for novel nanomedicine products. The evolving regulatory landscape and guidelines may introduce uncertainty and challenges during approval. Nonetheless, adhering to regulatory considerations is crucial to ensure the safe and successful development and commercialization of nanomaterial-based drug delivery systems [223].

## Conclusion

Significant advancements have been made in targeted and personalized pharmacotherapy, with promising potential in utilizing nanotechnology-based materials and devices. The development of nanoscale platforms for CNS disorders presents a particularly challenging and intricate task compared to traditional drug delivery methods. In addition to the requisite criteria of biocompatibility, biodegradability, biodistribution, precise pharmacokinetics and pharmacodynamics, optimal therapeutic outcomes, and minimal adverse effects, a nanostructured or nanoscale platform designed for CNS therapy must account for the distinctive attributes of brain tissue. The advancements in molecular and cellular biology, as well as modern biomedicine, have provided researchers with a comprehensive understanding of the intrinsic barriers present in the CNS, specifically focusing on the BBB. These barriers serve as natural protective structures in the human brain, safeguarding it against exogenous and endogenous molecules, including antigenic and therapeutic substances.

The potential of utilizing this technological modality for the management and detection of CNS pathologies has been a source of optimism. Polymer-based techniques and NMs are currently under investigation for the development of various nano solutions aimed at enhancing drug delivery to patients suffering from central nervous system disorders. In order to expedite the treatment of central nervous system-related ailments and promote progress in this field, a comprehensive understanding of various biomedical disciplines such as neuroscience, immunology, pharmacology, and molecular imaging, as well as materials science, biomaterials, and pharmaceuticals, including polymers, nanomaterials, drug, and genetics, is essential.
